# Prostate-specific membrane antigen (PSMA) assembles a macromolecular complex regulating growth and survival of prostate cancer cells “*in vitro*” and correlating with progression “*in vivo*”

**DOI:** 10.18632/oncotarget.12404

**Published:** 2016-10-03

**Authors:** Maria Elisa Perico, Silvia Grasso, Matteo Brunelli, Guido Martignoni, Enrico Munari, Enrico Moiso, Giulio Fracasso, Tiziana Cestari, Hassan Y. Naim, Vincenzo Bronte, Marco Colombatti, Dunia Ramarli

**Affiliations:** ^1^ Department of Pathology and Diagnostics, Section of Immunology, University of Verona, Verona, Italy; ^2^ Department of Pathology and Diagnostics, Section of Pathology, University of Verona, Verona Italy; ^3^ Department of Physiological Chemistry, University of Veterinary Medicine of Hannover, Hannover, Germany; ^4^ Department of Diagnostic Pathology, Azienda Ospedaliera Universitaria Integrata, Verona, Italy; ^5^ Department of Molecular Biotechnology and Health Sciences, University of Torino, Torino, Italy; ^6^ Current address: Department of Molecular Biotechnology and Health Sciences, University of Torino, Torino, Italy; ^7^ Current address: Department of Pathology, Pederzoli Hospital, Verona, Italy

**Keywords:** PSMA, castration-resistant prostate adenocarcinoma, p130CAS, BCAR1, phospho-EGFR receptor

## Abstract

The expression of Prostate Specific-Membrane Antigen (PSMA) increases in high-grade prostate carcinoma envisaging a role in growth and progression. We show here that clustering PSMA at LNCaP or PC3-PSMA cell membrane activates AKT and MAPK pathways thus promoting proliferation and survival. PSMA activity was dependent on the assembly of a macromolecular complex including filamin A, beta1 integrin, p130CAS, c-Src and EGFR. Within this complex beta1 integrin became activated thereby inducing a c-Src-dependent EGFR phosphorylation at Y^1086^ and Y^1173^ EGF-independent residues. Silencing or blocking experiments with drugs demonstrated that all the complex components were required for full PSMA-dependent promotion of cell growth and/or survival in 3D culture, but that p130CAS and EGFR exerted a major role. All PSMA complex components were found assembled in multiple samples of two high-grade prostate carcinomas and associated with EGFR phosphorylation at Y^1086^. The expression of p130CAS and pEGFR^Y1086^ was thus analysed by tissue micro array in 16 castration-resistant prostate carcinomas selected from 309 carcinomas and stratified from GS 3+4 to GS 5+5. Patients with Gleason Score ≤5 resulted negative whereas those with GS≥5 expressed p130CAS and pEGFR^Y1086^ in 75% and 60% of the cases, respectively.

Collectively, our results demonstrate for the first time that PSMA recruits a functionally active complex which is present in high-grade patients. In addition, two components of this complex, p130CAS and the novel pEGFR^Y1086^, correlate with progression in castration-resistant patients and could be therefore useful in therapeutic or surveillance strategies of these patients.

## INTRODUCTION

Prostate cancer (PCa) is the most common neoplasia and the third cause of cancer-related death in males in developed countries. Increasing numbers of patients benefit from early diagnosis, yet, the prognosis of the 5-6% of patients bearing advanced, castration resistant PCa remains unsatisfactory due to the failure of chemo- and radiotherapy regimens and the lack of early signatures of progression [1]. Pre-clinical and clinical evidence show that almost all PCa express PSMA, a transmembrane folate-hydrolase/carboxypeptidase involved in cellular nutrient uptake, which increases progressively in high-grade or castration resistant PCa *in vivo* [2, 3]. The relevance of PSMA as a diagnostic and prognostic marker is well established and its expression and function in neoplastic neo-angiogenesis has also pointed to the molecule as a therapeutic target [4–6]. Together with PSMA fresh PCa specimens may display a bio-molecular phenotype promoting survival and proliferation owing to a constitutive activation of the PI3K/AKT/mTOR and/or RAF/MEK/ERK pathways, an overexpression of p130CAS (also called BCAR1), a major scaffolding protein of the beta1 integrin (beta1) signalling platform, and an activation of beta1 itself. Noteworthy, p130CAS expression was correlated with PCa progression *in vivo* [3, 7–9].

Little information is available regarding activity of PSMA in regulating anti apoptotic pro-proliferative pathways eventually increasing resistance and aggressiveness of PCa cells. We have previously reported that clustering PSMA at the surface of LNCaP cells with specific monoclonal antibodies (mAbs), a treatment intended to mimic the PSMA encounter with its ligand(s), activates the RAS/RAC/MAPK pathway, NF-kB transactivation, IL-6 gene expression and CCL5 gene expression, further promoting the unlimited proliferation of LNCaP cells [10]. These findings prompted us to investigate whether PSMA clustering could activate also survival signalling and how the short PSMA cytodomain, lacking kinase or adaptor docking sites, could ensure transactivation. To this end we considered the possibility that filamin A (FLNa) a multi-domain cytoskeleton-associated protein binding both PSMA and beta1 cytodomains might anchor the two molecules thereby allowing a functional cooperation overcoming the PSMA structural inability to assemble signalling platforms. We further hypothesized that clustering PSMA-FLNa-beta1 in a macromolecular complex may surrogate adhesion thereby inducing beta1 activation, the association of beta1 signalling platform and the consequent relationship with growth factors such as EGFR [11–13].

We explored these hypothesis in prostate cancer cell lines, fresh specimens of PCa and paraffin embedded samples of patients with castration resistant high grade PCa.

## RESULTS

### PSMA cross-linking activates AKT/mTOR/BAD pathway and p38 and ERK1/2 MAPKs in LNCaP and PC3-PSMA cells

**Figure 1:**
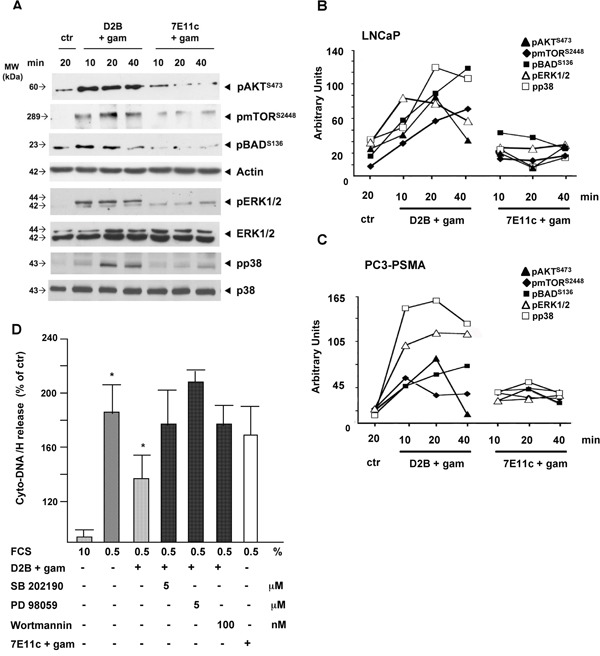
PSMA cross-linking activates AKT and MAPK pathways in the same cell population thereby counteracting apoptotic stimuli **A.** Crude LNCaP lysates subjected to D2B (D2B+gam) or 7E11c (7E11c+gam) cross-linking immunoblotted with the indicated mAbs. Actin, anti total p38 or ERK1/2 ensured equal loading in the lanes. **B, C.** Diagrams of pixel quantification of LNCaP (B) or PC3-PSMA (C) blots generated with ImageJ (http://imagej.nih.gov/ij/, 1997-2011). Symbols are as follows: filled rhomboids (mTOR^S2448^), filled squares (BAD^S136^), filled triangles (AKT^T473^), open triangles (pERK1/2), open squares (pp38). Values are the mean±SD of area pixels obtained in at least three independent experiments with p ranging 0.05-0.005 for LNCaP and 0.05-0.001 for PC3-PSMA. Treatment with 7E11c+gam did not provide significant results. **D.** Quantification of cyto-DNA/H release in LNCaP serum starved for 48 h and then subjected to D2B+gam cross-linking in the presence or absence of suboptimal doses of the indicated inhibitors. The 7E11c+gam was used as control cross-linking. Inhibitors were maintained throughout the experiment. OD values of three independent experiments were expressed as the percentage of untreated samples.

Results shown in Figure 1A demonstrated that PSMA-crosslinking fully activates the mTOR/AKT/BAD and the MAPK pathways in LNCaP cells, as revealed by using mAbs recognizing site-specific phosphorylation of AKT and mTOR (identifying the activation loop required for full activation of AKT) or mAbs recognizing the dual phosphorylation of the motif maximizing the catalytic activity of ERK1/2 p38 MAPK [14–16]. The basal activation of mTOR, AKT and BAD of untreated LNCaP cells was increased 2- to 3-fold after 10 min of treatment, it peaked at 20 min and decreased at 40 min. The activation of mTOR persisted up to 40 min. ERK1/2 and p38 activation followed similar kinetics in the same lysates. BAD phosphorylation was detected on Ser^132^, rather than on S^136^, suggesting that BAD lies downstream AKT rather than ERK1/2 activation. Similar results were obtained with PC3-PSMA cells (blots not shown). Pixel densitometry showed the significance of results obtained with LNCaP or PC3-PSMA cells (Figure 1B and Figure 1C, respectively), despite differences in the extent and/or the kinetics of activation. The cross-linking of 7E11c used as control, failed to exert any significant activity. In agreement with this, PSMA cross-linking rescued LNCaP cells from apoptosis induced by serum starvation (Figure 1D). The cytosolic release of DNA-histones, a marker of irreversible apoptosis, almost doubled in starved cells (192±28%). PSMA, but not 7E11c-cross-linking alleviated apoptosis to 130±27% (p<0.05). This rescue was abrogated by inhibiting p38 (SB, 179±56%), or ERK1/2 (PD, 217±7%) or AKT pathway (Wortmannin, 179±40%) at doses lacking apoptotic activity (Supplementary Figure S1), thus clearly demonstrating that PSMA rescue depended on the activation of both AKT and MAPK pathways although that of ERK1/2 seemed predominant if considering the stronger and more reproducible inhibition observed with PD (211%±6.7).

### PSMA cross-linking induces the association of FLNa, beta1, phospho-p130CAS, phospho-c-Src and EGFR in a macromolecular complex regulating the activation of AKT and ERK

**Figure 2:**
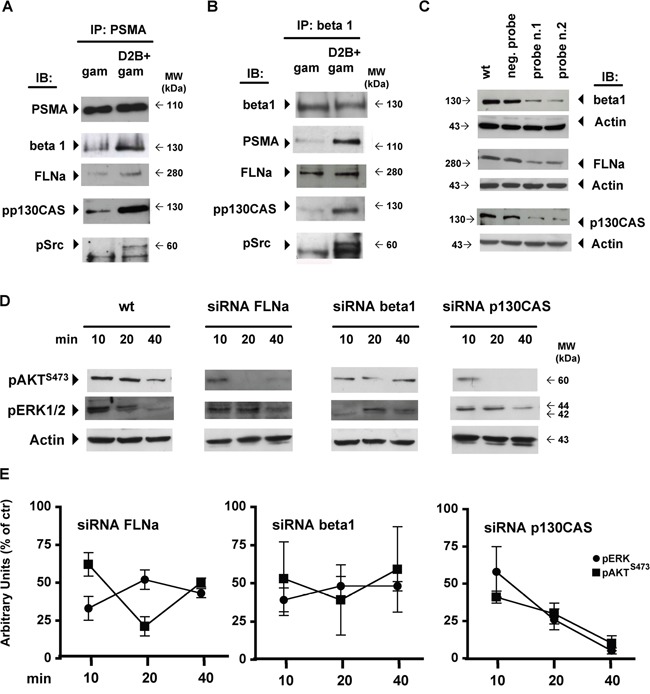
PSMA cross-linking triggers the assembly of a macromolecular complex regulating AKT and ERK1/2 activation Immunoprecipitation (IP) of PSMA **A.** or beta1 **B.** prepared from D2B+gam or gam treated LNCaP lysates and immunoblotted (IB) with mAbs as indicated by arrows. **C.** Immunoblotting of crude lysates of wild type LNCaP cells (wt) or siRNA silenced for beta1, FLNa, or p130CAS. Activity of relative negative or positive probes is indicated. **D.** Representative immunoblotting out of three performed of crude lysate prepared from wt, siRNA FLNa, siRNA beta1 or siRNA p130CAS silenced LNCaP cells probed with anti pERK1/2 or anti pAKT mAbs at the indicated time points after D2B+gam treatment. **E.** Diagrams of mean values± SD of pixel densitometry calculated as the percentages of those of wt cells at the same time points and expressed as residual activity at 10, 20 and 40 min. p value ranged from <0.05 to <0.01 at all points, except for pAKT in siRNA FLNa cells at 10 min.

As shown in Figure 2A PSMA assembled a large macromolecular complex when cross-linked. The low amount of beta1, FLNa and pp130CAS co-immunoprecipitated with PSMA in gam-treated LNCaP cells increased substantially upon D2B cross-linking, dramatically in the case of pp130CAS. Phospho-c-Src was undetectable in gam-treated cells and clearly detectable after PSMA cross-linking. Cross-immunoprecipitation of beta1 from the same lysates showed the presence of PSMA (Figure 2B) together with FLNa, pp130CAS and phospho c-Src.

Similar results were obtained by replacing LNCaP with PC3-PSMA cells in the same experimental setup and equal amounts of LNCaP or PC3-PSMA cell lysates were used for immunoprecipitation (Supplementary Figure S2). SiRNA silencing was used to assay the requirement of FLNa, beta1 and p130CAS for AKT or ERK1/2 activation. Representative IBs shown in Figure 2C demonstrated that the matched probes (Pos. probe n.1, n.2), but not mismatched ones (Neg. probe), significantly reduced the expression of FLNa (69±5%), beta1 (62±7.5%) or p130CAS (58±12%). No effect was observed on the membrane expression of PSMA which was 98.1%±1.5 in siRNA FLNa cells and 94.5±.9 siRNA beta1 cells (not shown). A stronger silencing induced substantial anoikis in the cells and it was therefore avoided. Even under these suboptimal conditions, the phosphorylation of AKT and ERK1/2 induced by PSMA cross-linking was largely reduced at all points in FLNa- or beta1-silenced cells (Figure 2D, Figure 2E). The greatest reduction was observed silencing p130CAS. Residual activity was less than 25% of that of wild type cells (wt) at 20 min and nearly abrogated at 40 min thus putting into evidence the hierarchical role of p130CAS in the AKT or ERK1/2 activation induced by the PSMA macromolecular complex.

### PSMA macromolecular complex regulates growth and/or apoptosis resistance of LNCaP in 3D culture

**Figure 3:**
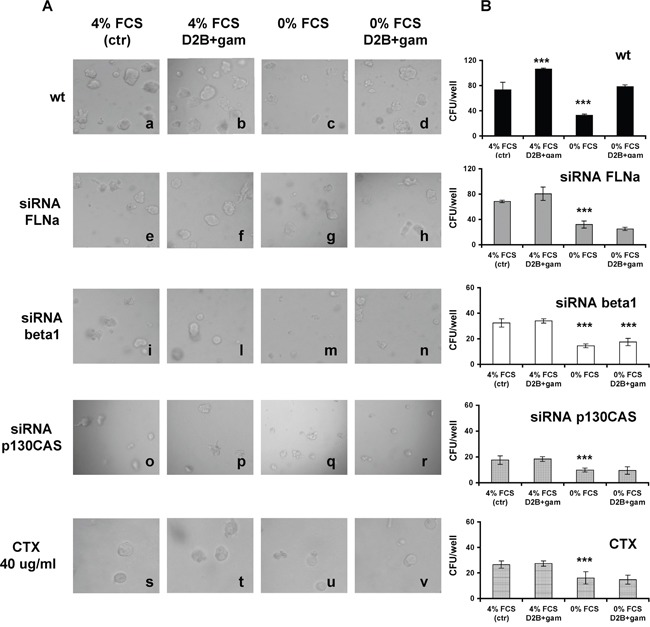
FLNa, beta1, p130CAS silencing or treatment with Cetuximab (CTX) hamper LNCaP cells growth in 3D cultures and abrogates the promoting or rescuing ability of PSMA cross-linking **A.** Representative phase contrast images (20X magnification) of colonies grown from wt, or siRNA silenced LNCaP cells or Cetuximab treated LNCaP cells cultured as indicated at 4% FCS (a, e, i, o, s) or at 4% FCS plus D2B cross-linking (b, f, l, p, t) or at 0% FCS (c, g, m, q, u) or at 0% FCS plus D2B+ cross-linking (d, h, n, r, v). **B.** Mean values±SD of colonies counted in three independent experiments performed in triplicate with the indicated LNCaP cell populations. p values are calculated comparing results with the control of the group of treatment.

As shown by images and diagrams presented in Figure 3A and Figure 3B, wt LNCaP cells form colonies (CFU) when cultured in Matrigel at 4% FCS (73.3±12/well, a). CFU number increased upon PSMA cross-linking (106±2,b), dropped to 32.7±2.1 in the absence of serum (0% FCS) showing the “shrinking” phenotype of apoptotic cells (c) and it was fully restored by PSMA cross-linking (78.3±3, d). Silencing FLNa slightly diminished the CFU growth at 4% FCS (68.5±2, e) or the proliferation induced by PSMA cross-linking (80.5±10.5,f), but it abrogated the rescuing activity of PSMA cross-linking (25±2.6, h). Silencing beta1, or p130CAS, had a powerful impact on LNCaP behaviour. The CFU developing under standard conditions were reduced to 32.5±3.3 (i) or to 17.5±1.5(o) respectively, dropping to 14.5±1.5 (m) and 9.8±0.8 (q) in the absence of serum. PSMA cross-linking failed to promote proliferation (l, p) or to rescue serum starved cells (n, r). Very similar results were obtained inhibiting EGFR function with Cetuximab (CTX) (s-v). The cross-linking of 7E11c was devoid of activity (data not shown) thus confirming that the PSMA macromolecular complex regulates growth and resistance to apoptosis in LNCaP cells depending on the FLNa-mediated association with beta1 and p130CAS. Similar results were obtained when LNCaP cells were replaced with PC3-PSMA cells in the same experimental setup (Supplementary Figure S3)

### PSMA cross-linking induces beta1 activation and EGFR phosphorylation at EGF-distinct residues

**Figure 4:**
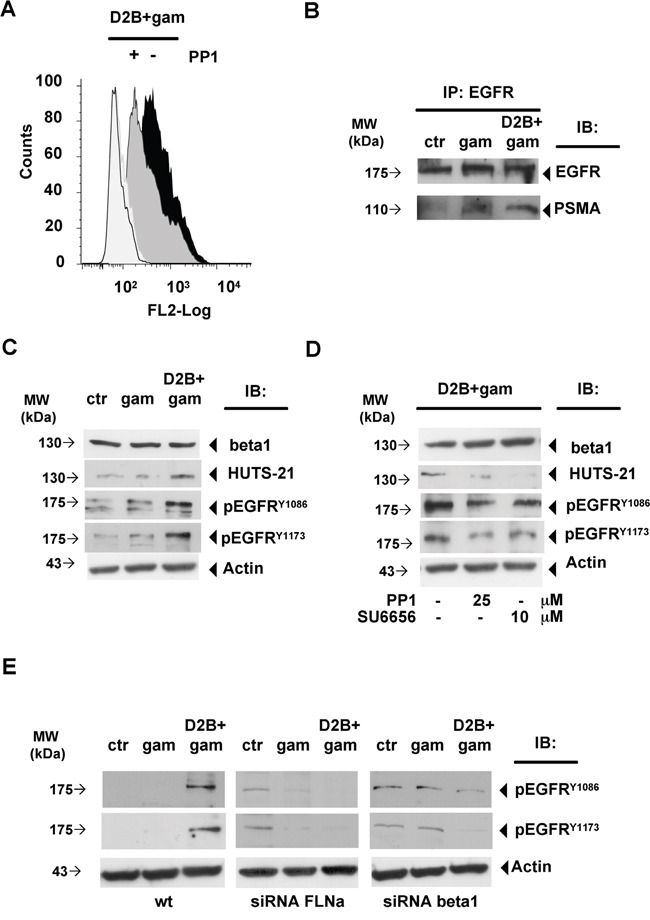
PSMA cross-linking induces beta1 activation and phosphorylation at Y1086 and Y1173 of EGFR associated to the complex **A.** Flow cytometry of HUTS-21 expression in LNCaP cells left untreated (empty histogram), treated with D2B+gam (black histogram) or treated with D2B+gam in the presence of PP1 (grey histogram). Isotype control is depicted. **B.** Representative immunoblotting of EGFR-IP of LNCaP cells left untreated (ctr) or subjected to gam- or D2B+gam cross-linking. PSMA pulled out band is indicated. Equal amounts of cell lysate were immunoprecipitated (Supplementary Figure S2) **C.** Immunoblotting of crude lysates of LNCaP cells treated as in B and probed with anti HUTS-21, anti pEGFR^Y1086^ and anti pEGFR^Y1173^ mAbs. **D.** Immunoblotting of LNCaP cells treated as in B in the presence or absence of PP1 or SU665 at the indicated doses. **E.** Immunoblotting of LNCaP wt cells, FLNa or beta 1 silenced LNCap cells as in B and probed with anti pEGFR^Y1086^ and anti pEGFR^Y1173^ mAbs. Results are representative of at least two experiments. Equal amounts of cell lysates were used for EGFR IP (Supplementary Figure S2). Arrows indicate relevant bands in all panels.

As previously mentioned activated beta1 associates with p130CAS, c-Src and EGFR leading to EGFR transactivation at EGF-distinct residues such as Y^1086^ and Y^1173^ [11–13]. As shown in Figure 4A, activated beta1 was almost undetectable in LNCaP cells untreated (0.3%±0.2, mean fluorescence, m.f. 83.5±5.5) or treated with gam (0.4%±0.2, m.f. 124±5, not shown). PSMA cross-linking rapidly induced a strong activation of beta1 which exposed the HUTS-21 epitope on 62% of the cells (62%±0.4, m.f. 630 ±1.5). Cross-linking of 7E11c was devoid of activity (2.3%±0.2, m.f. 128±4, not shown) thus indicating that beta1 activation was dependent on interactions between its own and PSMA cytodomain. The involvement of c-Src requirement was demonstrated by the strong reduction of HUTS-21 positive cells (45%±1.3, m.f. 358±6) determined by c-Src inhibitor PP1, used instead of the more specific SU6656 [17] because of the unexpected strong autofluorescence of the latter. The finding that EGFR associates with PSMA after cross-linking (Figure 4B), as part of the macromolecular complex described in Figure 2, prompted us to examine whether it was phosphorylated. Immunoblotting shown in Figure 4C and Figure 4D demonstrated that PSMA cross-linking induced activation of beta1 and, in the same cell lysates, phosphorylation of EGFR at Y^1086^ and Y^1173^ residues. PP1 or SU6656 abrogated beta1 activation meanwhile strongly decreasing EGFR phosphorylation at Y^1086^ and Y^1173^ residues as well as the silencing of FLNa or beta1 (Figure 4E). Gel densitometry of Figure 4C, Figure 4D and Figure 4E is provided in Supplementary Figure S4. As previously reported, the basal phosphorylation of EGFR^Y1086^ and EGFR^Y1173^ was found increased in silenced cells, most likely depending, as reported in other experimental systems, on an increased EGFR recycling which did not alter its reactivity to the ligand [12].

Collectively, these results clearly demonstrate that EGFR transactivation lied downstream a chain of events starting with PSMA clustering at the cell surface and for this it is likely to be considered as a signature of a functionally active complex.

### PSMA macromolecular complex is detected “*in vivo*” where it correlates with PCa progression

**Figure 5:**
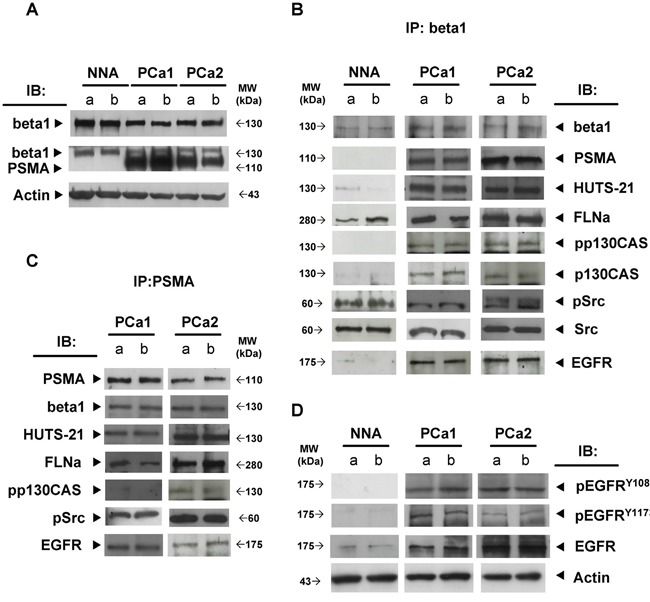
PSMA macromolecular complex is assembled “*in vivo*” **A.** Beta 1 and PSMA expression in NNA and PCa tissue lysates. Beta1 **B.** or PSMA **C.** IP prepared from NNA and PCa tissue lysates. Co-immunoprecipitated proteins are detected by immunoblotting as indicated **D.** Immunoblotting of NNA and PCa tissue lysates probed with anti EGFR, anti pEGFR^Y1086^ and anti pEGFR^Y1173^ mAbs. Actin shows loading in A and D.

**Figure 6:**
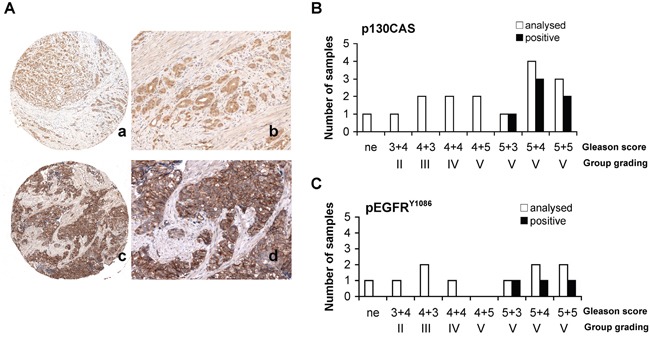
TAM immunohistochemistry **A.** Representative immunohistochemistry of TMA showing high score (2+) for p130CAS (a, b) and pEGFR^Y1086^(c, d). Magnification is as follows: 4X (a, c), 20X (b, d). p130CAS high score: ≥30% of the cells stained strongly, low score: ≥10% of the cells stained weakly or <30% stained strongly. pEGFR^Y1086^ high score: ≥10% of cells stained strongly, low score: >10% of the cells stained weakly **B, C.** Results of TAM immunohistochemistry for p130CAS (B) (16 patients) and pEGFR^Y1086^ (C) (10 out of 16 patients) with castration resistant prostate adenocarcinoma. In uppercase H and L indicate high and low score respectively, “a” indicates samples positive for both p130CAS and pEGFR^Y1086^, “ne” indicates neuroendocrine. p130CAS expression was scored as 2+), or as 1+) or as 0 (negative). pEGFR^Y1086^ expression was scored as 2+), or as 1+).

We next investigated whether the PSMA macromolecular complex could be found assembled “*in vivo*” in frozen prostate specimens obtained from two patients with GS 8 (PCa1, PCa2). Two prostate specimens (GS 6) sampled in the 90% remaining non neoplastic area (NNA) was used as control. Each specimen was cut in four pieces and two opposite pieces analysed (a and b). PSMA was not expressed in the representative NNA (Figure 5A) whereas it was strongly expressed in both samples of PCa1 and PCa2. FLNa, phospho c-Src and a minute amount of activated beta1 (HUTS-21) were co-immunoprecipitated with beta1 in the NNA samples (Figure 5B) whereas PSMA, FLNa, pp130CAS, phospho c-Src, activated beta1 and EGFR were co-immune- and cross-immunoprecipitated in both samples of PCa1 and PCa2 (Figure 5B and Figure 5C). The presence of activated beta1 in the PSMA macromolecular complex led us to investigate whether EGFR was phosphorylated at Y^1086^ and Y^1173^ residues. As shown in Figure 5D, pEGFR^Y1086^ and pEGFR^Y1173^ were not detectable in the NNA lysate, but they were clearly present in all 4 samples of PCa1 and PCa2 although in different amounts. The additional NNA specimen (NNA2) was obtained, treated and analysed as NNA. Results of immunoblotting of separate opposite fragments and corresponding IPs resembled those of NNA are shown in the Supplementary Figure S5. Taken together the biochemical analysis of PCa tissues demonstrated that the PSMA macromolecular complex was present and functionally active in the PCa specimens examined. The potential correlations between the assembly of PSMA molecular complex and PCa progression were investigated by immunohistochemistry of Tissue Macro Arrays (TMA) of castration resistant PCa from 16 patients (15 adenocarcinoma and 1 neuroendocrine carcinoma) selected from 309 patients stratified according to GS and group grading (Table 1). Representative images of a high expression of p130CAS or of pEGFR^Y1086^ are respectively shown in Figure 6A (a,c and b,d, respectively). The results of the screening (Figure 6) demonstrated that both p130CAS (Figure 6B) and pEGFR^Y1086^ (Figure 6C) expression correlates with GS and therefore with progression. Notably, positive staining of p130CAS was observed only in the eight patients with primary GS >5 (thus combined ≥5) whereas the eight patients scoring primary GS< 5 were negative so that the overall positivity of 37% raised to 75% if considering the primary GS>5 patients only. The pEGFR^Y1086^ expression was evaluated on the remaining serial sections TMA slides of 10 patients. Similarly to p130CAS the positive staining was restricted to primary GS>5 and the 30% overall positivity increased to 60% in the highly graded patients. Moreover, it must be noticed that only 2 out of the 3 pEGFR^Y1086^ positive cases shared p130CAS positivity whereas the third showed pEGFR^Y1086^ only. This finding may mirror the scattered detection of pEGFR^Y1086^/ pEGFR^Y1173^ in PCa tissue fragments, most likely due to variable intensities of signalling foci. For this, it suggests that pEGFR^Y1086^ may be included among the few markers available nowadays of progression of PCa.

**Table 1:** Pathological characteristics of the study population. Data retrospectively reviewed from 309 consecutive patients who underwent radical retropubic prostatectomy or laparoscopic robot-assisted prostatectomy

		Routinely available Prostate Carcinoma (N=309)				Metastatic Prostate Carcinoma	Castration Resistant Prostate Carcinoma
number of samples	%	Gleason Score	Group Grading	number of samples	%	number of samples	number of samples
90	29%	3+3	I	88	28%		
146	47%	3+4	II	141	45%		
25	8%	4+3	III	28	10%		
27	9%	4+4	IV	30	10%	4	**16 (5%)**
21	7%	9, 10	V	22	7%	12	

**Figure 7:**
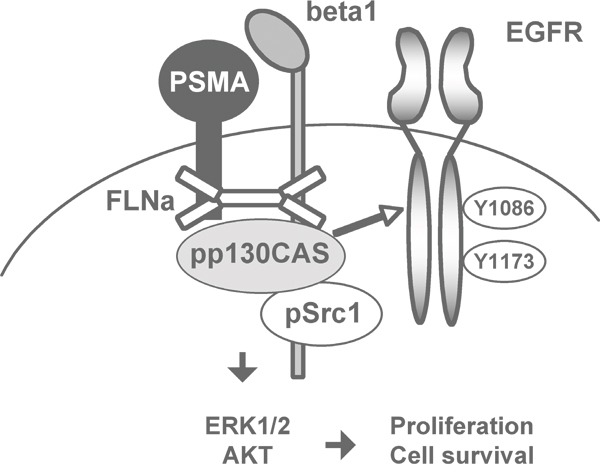
Cartoon illustrating a visual summary of results

A cartoon summarizing the results of the biochemical investigations performed “*in vitro*” and “*in vivo*” on the relationships entertained by the components of the PSMA macromolecular complex provides a visual summary of the model we propose (Figure 7).

## DISCUSSION

In this study we provide novel information on the role of PSMA in the mechanisms of prostate cancer cell growth and progression. Results obtained “*in vitro*” demonstrate that PSMA clustering induces: 1) the PSMA association to FLNa in a macromolecular complex including beta1, phospho-p130CAS, phospho c-Src and EGFR 2) the activation of beta1, the phosphorylation of c-Src and thereby the transactivation of EGFR at EGF-independent Y^1086^ and Y^1173^ residues 3) a burst of proliferation and resistance to apoptosis sustained, among other mechanisms, by the activation of the AKT/mTOR/BAD and MAPK pathways. The possibility that results obtained “*in vitro*” could be merely observed in cell lines derived from selected clones of metastatic PCa such as LNCaP or PC3-PSMA was ruled outhe finding that: 1) the macromolecular complex including PSMA, FLNa, activated beta1, phospho p130CAS, phospho c-Src and phospho EGFR^Y1086^ or phospho EGFR^Y1173^ was co-immunoprecipitated from human tissues of high graded PCa, but not from non neoplastic areas and 2) a high expression of p130CAS or pEGFR^Y1086^ was detectable by TMA immunohistochemistry in patients with castration resistant PCa only when primary GS was five or above.

Two considerations can be made based on the consistency between “*in vitro*” and “*in vivo*” observations. The first involves the presence of natural ligands of PSMA in the neoplastic prostate. In particular, the presence of folate has ben reported to provide a growth advantage to cancer cells [18]. The second concerns the likelihood that the promoting activity on cell growth and survival induced by PSMA clustering “*in vitro*” may occur also “*in vivo*”.

In our experimental system FLNa and p130CAS had the greatest relevance among the components of the PSMA macromolecular complex: the first in the core association of PSMA with beta1, the second in the chain of signalling events eventually regulating the downstream biological activities. FLNa is a well known cytoskeletal-associated protein binding a variety of partners and playing different roles in cancer according to its subcellular localization. Cytoplasmic FLNa has a cancer-promoting activity due to multiple interactions with signalling proteins. In agreement with the expression and the cytoplasmic localization of FLNa in PCa cells FLNa itself has been reported to increase and correlate with an invasive phenotype [19, 20]. By contrast, nuclear FLNa has been shown to suppress cancer growth by interacting with transcription factors [20, 21].

We examined the molecular interaction of cytoplasmic FLNa, more specifically, the interactions occurring between transmembrane or iuxta-membrane proteins. Dependent on this subcellular localization the FLNa recognition of specific binding sites for PSMA and beta1 could be favoured by the co-localization of the three molecules in the same lipid rafts, known to behave as privileged platforms for membrane sorting, trafficking and signalling in normal and cancer cells [22, 23]. In fact, the analysis of the distribution of PSMA partners in Lubrol lipid rafts (Supplementary Figure S6) demonstrated that PSMA, beta1 and FLNa are already located in the same Detergent Resistant Membranes (DRMs) at steady state in LNCaP cells. By contrast, p130CAS and c-Scr were exclusively found in DRMs if phosphorylated (ibidem). According to this, FLNa, PSMA and beta1 were among the molecules that were co-immunoprecipitated in untreated or gam-treated LNCaP or PC3-PSMA cells. PSMA clustering strongly increased the number and/or the amount of molecules in the basal complex, particularly phospho-p130CAS, thereby inducing c-Src phosphorylation and association. It can be therefore envisaged that PSMA clustering could promote p130CAS and c-Src displacement from the soluble milieu to DRMs thereby favouring a circuit of progressive assembly of increasing amounts of PSMA macromolecular complexes. Functional studies supported the key role of FLNa in the assembly of an active PSMA macromolecular complex as FLNa silenced LNCaP were not rescued by PSMA clustering in 3D cultures. Similarly, phosphorylation of EGFR^Y1086^ or EGFR^Y1173^ was not induced upon beta1 activation.

A great deal of interest has been paid to dissecting the role of the components of the beta1 signalling platform in the growth and the invasion of cancer including that of prostate [8, 9, 11-13, 24, 25]. In our experimental system beta1 was found activated when complexed with FLNa and PSMA both in “*vitro*” and “*in vivo*”. Previous reports have shown that FLNa inhibits the inside-out activation of beta1 unless it is displaced by migfilin, as in endothelial cells, or by talin, as in fibroblasts, and that talin1 is required for beta1 activation in PCa cell lines selected to promote bone metastasis in mice [26–28]. The contrast with our results is only apparent because neither endothelial cells nor fibroblasts express PSMA. As regards beta1 activation in PCa cell lines the mechanisms of beta1 activation described by Jin et al (2015) in previous reports and in our study are not mutually exclusive. No recognition sequence for talin1 has been described in PSMA cytodomain. Thus, it is conceivable that beta1 may either bind to talin1 or to FLNa and that the two mechanisms may account for the overall beta1 activation observed in primary and metastatic PCa “*in vivo*” [25]. The finding that beta1 activation is inducible in HUVECs by an outside-in signal depending on the processing activity of PSMA and MMP-2 of laminin peptides adds in our scenario the increase of angiogenesis required for the prostate cancer to grow [6].

Our previous and herein shown results demonstrate that PSMA macromolecular complex regulates pivotal processes of LNCaP cell growth such as proliferation and survival. This occurs due to several mechanisms: the activation of RAS-MAPK and AKT-BAD pathways and of NF-κB transcription factor, the gene expression of IL-6 and CCL5 [9], the activation of beta1 integrin, the consequent phosphorylation of EGFR at Y^1086^ and Y^1173^ residues which is strictly correlated with protection from apoptosis [11].

SiRNA silencing experiments clearly demonstrated the link between AKT and MAPK phosphorylation, PSMA-mediated rescue and assembly of PSMA macromolecular complex. However, silencing p130CAS had the most dramatic effects both on AKT and ERK1/2 activation, on LNCaP cells growth and on the response to apoptosis in 3D assays. This is in line with recent reports correlating the expression of p130CAS with PCa aggressiveness on the basis of 15% positive low-grade localized PCa, 48% positive high-grade localized tumours, 60% positive lymph node metastases and 80% positive castration-resistant PCa [29]. This observation also agreed with our results “*in vivo*” showing phospho p130CAS complexed with PSMA in high grade PCa and castration resistant patients. Based on “*in vitro*” and “*in vivo*” results we proposed a model of PSMA activity which is depicted in the cartoon of Figure 7 and it links proliferation and cell survival to the assembly of Filamin A, beta1 integrin, p130CAS, c-Src and EGFR in a signalling complex where beta1 integrin becomes activated thereby inducing a c-Src-dependent EGFR phosphorylation at EGF-independent residues (Y^1086^ and Y^1173^). We therefore decided to probe a panel of castration resistant patients with mAbs anti pEGFR^Y1086^. Results of TMA immunohistochemistry demonstrated that 3/3 patients with primary GS at least 5 and combined higher than 5, (Grade Groups 5, five-grade group system, by Epstein et al.) [30, 31], expressed pEGFR^Y1086^ however only two of them share p130CAS expression whereas the third was positive only for pEGFR^Y1086^.

We are aware that our panel of castration resistant patients, although very carefully analysed by TMA immunohistochemistry and tightly monitored is not sufficiently large to provide statistically significant results, however, it has to be noted that this group accounts for the 5-6% of patients only and even wide databases such as the Cancer Genome Atlas (TCGA platform) host a number of castration resistant patients even lower than ours being therefore unsuitable for significant negative or positive association studies. This may strengthen the value of our observation regarding the expression of p130CAS and pEGFR^Y1086^ which could be both inserted in nomograms useful in selecting patients for “active surveillance” versus aggressive surgical or medical approaches.

## MATERIALS AND METHODS

### Cells and cell treatment

The LNCaP, purchased from the American Tissue Culture Collection Rockville Pike, MD, USA) and the PC3-PSMA (kindly provided by Dr. W. Heston, Cleveland Clinic Foundation, Cleveland, OH, USA) cell lines were cultured in RPMI at 10% FCS (EuroClone, Milano, Italy). LNCaP cells were plated on poly-D-lysine coated plasticware (10 μg/ml) (SIGMA Aldrich, St. Louis, MO, USA). PSMA clustering was induced by the binding of D2B mAb followed by the binding of (Fab)_2_ goat anti-mouse (Li StarFish, Milan, Italy) as previously described [10] and henceforth designated “PSMA cross-linking”. MAb D2B, recognizing an extracellular domain of PSMA was produced, characterized and purified in our laboratory [10]. MAb 7E11c (clone HB-10494, from ATCC) recognized an intracellular epitope of PSMA and replaced D2B as control. Apoptosis was induced by FCS starvation (0.5% FCS for 48 h). PSMA or 7E11c cross-linking was performed before and 24 h later than FCS reduction. Apoptosis was evaluated by measuring the cytosolic release of DNA/histone complexes (cyto-DNA/H assay) following the manufacturer's protocol (Cell Death Detection ELISAPlus, Roche, Mannheim, Germany).

The 3D colony formation assay was performed according to Debnath et al. [32] with minor modifications. BD Matrigel growth factor reduced (BD Biosciences, San Josè, CA, USA)(40 μl/well) was layered in flat-bottomed 96-well plates and allowed to solidify at 4°C. LNCaP cells (2.5×10^3^/well), plated in 200 μl of RPMI at 4% FCS and 3% Matrigel, were subjected to PSMA or mock cross-linking. At day 6-8 the cultures, fixed with 10% ethanol in H_2_O at 0.005% crystal violet were examined with a phase contrast optical microscope equipped with a gridded ocular lens. Clones measuring 0.150-0.5 mm were scored as a CFU.

### Human tissues and case selection

This study was approved by the AOUI ethical Committee (n.45767-Bioimm 2014). PCa specimens were obtained from three patients subjected to radical prostatectomy, immediately frozen and maintained at −80°C (ARC Net bio bank, Verona). A 5 μm tissue section was prepared from frozen specimens and a diagnosis of PCa confirmed by the pathologist. Paraffin embedded specimens were retrieved from 16 prostatectomy specimens of patients with castration resistant PCa from the archives of the University of Verona from 2003 to 2008 and reported with a clinical history of resistant deprivation therapy to androgens. Histological diagnosis was performed by expert pathologists (GM, MB, EM). Tissue microarray (TMA) was built with 5 array cores per case, measuring 0.6 mm in diameter each, after selecting representative areas of each tumor specimen and 2 normal prostate tissues.

### Immunoblotting (IB), Immunoprecipitation (IP), Immunohistochemistry (IHC) and Flow Cytometry

IB was performed according to standard techniques with cell lysates prepared as previously described and proteins quantified by Biorad protein assay reagent (Hercules, CA, USA). Samples were analysed by SDS-PAGE under reducing conditions and electroblotted to nitrocellulose membranes (Biorad, Hercules, CA, USA). Tissue lysates were prepared from frozen samples kept ice-cold. Tissues (35-45 mg) were minced, lysed in RIPA buffer with protein inhibitory cocktail and sodium orthovanadate 5 mM (Sigma-Aldrich), homogenized, sonicated, incubated for 30 minutes on ice and centrifuged for 30 min (14000 rpm at 4°C). Cleared supernatants were collected and used in IB or IP procedures. Membranes were probed with the following monoclonal or polyclonal Abs directed to: pp38, pAkt^Thr473^, pERK1/2^Thr202/Tyr204^, pBad^Ser136^, pmTOR^Ser2448^, p130CAS, pp130CAS, phospho c-Src, pSrc^Y416^, pEGFR^Y1086^, pEGFR^Y1173^ (Cell Signalling Technology, Danvers, MA, USA), FLNa from Bethyl (Montgomery, TX, USA), actin (Sigma-Aldrich), beta1 (clone BV7, Abcam, Cambridge, UK), EGFR (clone MINT-5, kindly provided by Dr. S. Canevari, Istituto Nazionale Tumori-Milano, Italy) [33]. HRP-conjugated anti-rabbit or anti-mouse IgG were from Millipore (Temecula, CA, USA). Signals were detected by ECL (Lite Abbot Extend or Turbo western blot detection kits) (Euroclone). Each IP was performed starting with 500 μg of pre-cleared lysates, mixed with the appropriate Ab, washed, immunoprecipitated with Protein G Sepharose 4 Fast Flow (GE Healthcare Biosciences AB, Uppsala, Sweden), and analysed by loading 20 μl in each lane (Replica lanes). Replicas were probed with the different Abs by IB under reducing conditions. IHC was performed on TMA slides immune labeled with anti p130CAS (1:100, pH 8.9, 30 min, poly 60) or anti pEGFR^Y1086^ Ab (1:50). Before the analysis on TMA, the anti pEGFR^Y1173^ and anti pEGFR^Y1086^ antibodies were subjected to validation. Briefly, LNCaP cells were activated, washed, centrifuged for 10 min at 1500 rpm, and resuspended in 75%methanol/ 20% chloroform/5% acetic acid for 10 min. Pellets were treated with formaldeheide for 30 min, washed twice with ethanol and paraffin embedded and routinely stained with antibodies at 1:50 and 1:100 dilution. Only the anti pEGFR^Y1086^ Ab showed diffuse immunoexpression on Cytocell inclusion and therefore used on TMA [34]. Heat-induced antigen retrieval was achieved heating the slides for 30 minutes in the presence of citrate buffer (10 mM, pH 6.0). Samples were then processed by using a “Bond Polymer Refine” detection system in an automated Bond immunostainer (Vision Biosystem, Menarini, Florence, Italy). Cytofluorometry was performed according to standard techniques by using a BD FACSCanto II (Becton-Dickinson, Frankin Lakes, NJ, USA).

### Kinase inhibition and siRNA silencing

AKT, ERK1/2 or p38 MAPKs were respectively inhibited by dose response treatment with Wortmannin or PD098059 or SB202190 drugs (Calbiochem-Novabiochem, San Diego, CA, USA); c-Src activity was inhibited by PP1 or SU6656 (Sigma-Aldrich). siRNA silencing was achieved by using the TriFECTa Kit DsiRNA duplex following the manufacturer's instructions (IDT Tech., Inc. Coralville, IA, USA). FLNa (accession code NM_001456), beta1 (NM_00221) or p130CAS (NM_001170714) oligoduplexes were used at 40, 200 or 100 nM, respectively. Mismatched oligoduplexes were used at a concentration of 50 nM. Silencing was evaluated by IB and flow cytometry performed at day 3-5 from treatment.

### Statistical analysis

Results are presented as the mean±SEM. Significance, assessed by using a two-tailed Student's test was expressed as follows: * p<0.05; ** p<0.01; and *** p<0.001.

## SUPPLEMENTARY MATERIALS FIGURES


