# Supplementary material for: Prostate-specific membrane antigen (PSMA) assembles a macromolecular complex regulating growth and survival of prostate cancer cells “in vitro” and correlating with progression “in vivo”
Source: Oncotarget. 2016 Oct 3;7(45):74189–202. doi: 10.18632/oncotarget.12404 (PMC5342045; doi:10.18632/oncotarget.12404)
Supplement: Supplementary file 1 [file oncotarget-07-74189-s001.pdf]

Prostate-specific membrane antigen (PSMA) assembles a macromolecular complex regulating growth and survival of prostate cancer cells "*in vitro*" and correlating with progression "*in vivo*"

SUPPLEMENTARY FIGURES

A

SB 202190 (μM)	-	5	-	5	-	5	25	25	25
PD 98059 (μM)	-	5	-	5	-	5	25	25	25
Wortmannin (nM)	-	-	100	100	200	200	-	100	200
% of positive cells	2.5±1.5	6.4±3.2	6.4±2	6.2±2.8	7.8±1.3	11.2±5.5	13.6±6.2	13.6±5.5	30±8.5
p<		ns	ns	ns	0.05	0.05	0.05	0.001	0.001

B

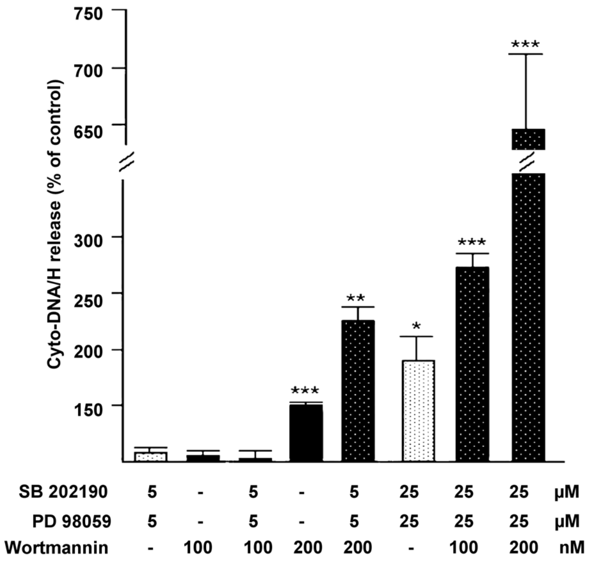

Supplementary Figure S1: Effect of MAPK or AKT inhibition on spontaneous apoptosis of LNCaP cells. A. Mean results±SD of Annexin V staining on LNCaP treated with SB202190, PD98059 or Wortmannin. **B.** cytoDNA/H quantitation in the same experimental set up. OD values of three independent experiments were expressed as the percentage of untreated samples.

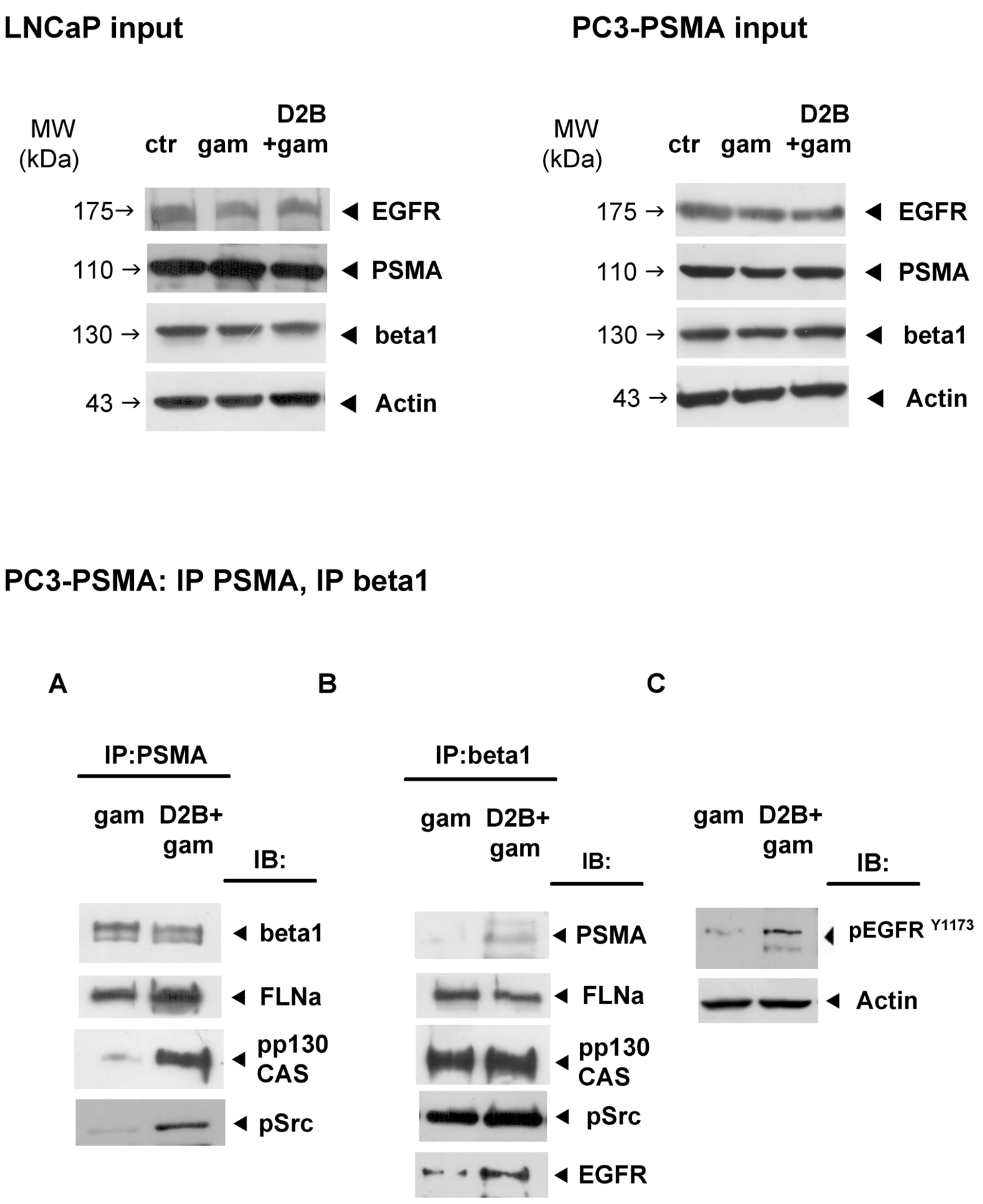

Supplementary Figure S2: PSMA assembles a similar molecular complex in LNCaP and PC3-PSMA cells. Upper panels: equal amounts of LNCaP and PC3-PSMA lysate are used for Immunoprecipitation. **A, B.** Immunoprecipitation (IP) of PSMA (A) or beta1 (B) prepared from D2B+gam or gam treated PC3-PSMA lysates. **C.** Immunoblotting (IB) of crude lysates of PC3-PSMA, treated with D2B+gam or gam. Arrows indicate mAbs used for immunoblotting.

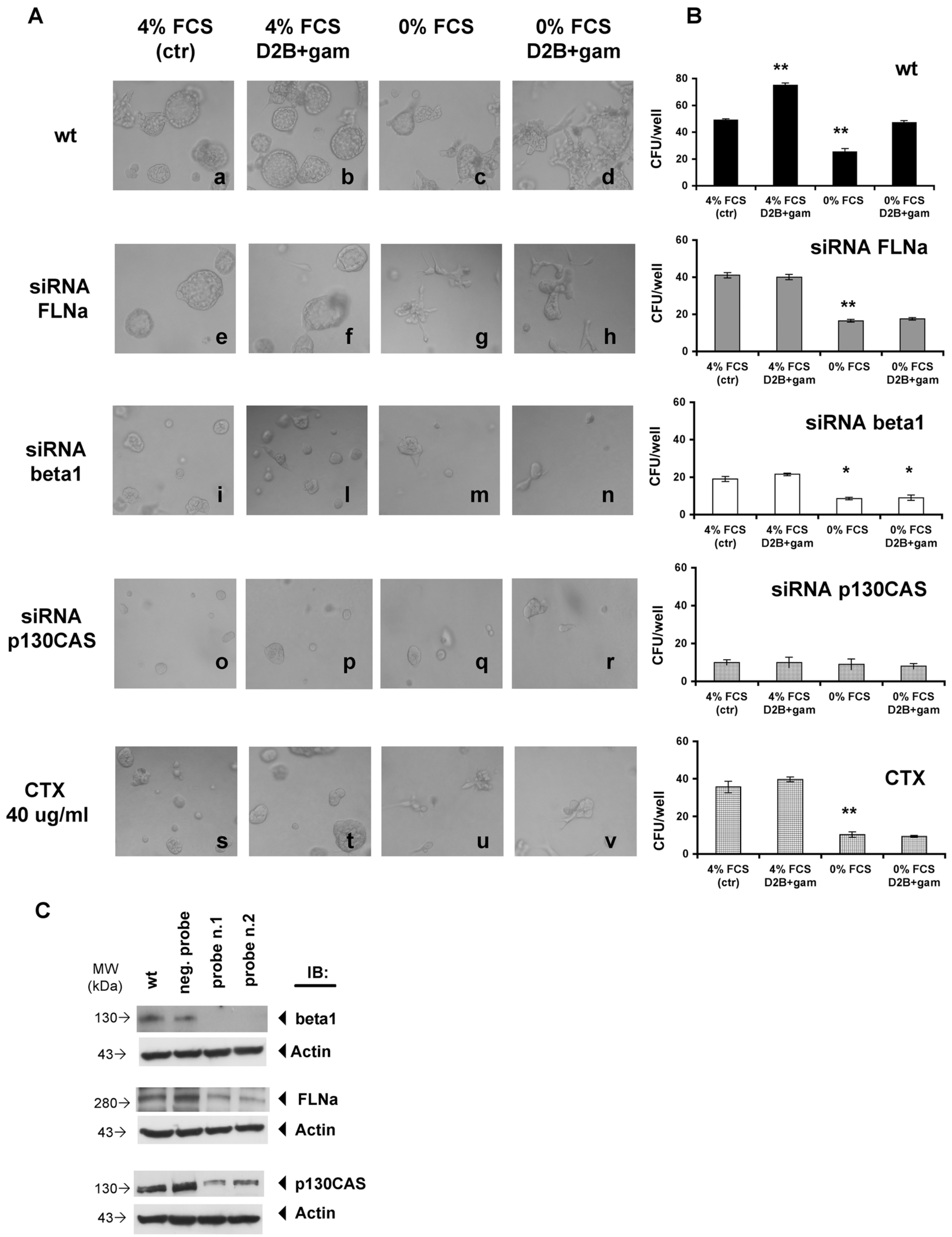

Supplementary Figure S3: FLNa, beta1 or p130CAS silencing or Cetuximab (CTX) treatment hamper PC3-PSMA cell growth in 3D cultures and abrogate the promoting or rescuing ability of PSMA cross-linking. A. Representative phase contrast images (20X magnification) of colonies grown from wt or siRNA silenced PC3-PSMA or Cetuximab treated PC3-PSMA cells as indicated cultured at 4% FCS (a, e, i, o, s) or at 4% FCS plus D2B cross-linking (b, f, l, p, t) or at 0% FCS (c, g, m, q, u) or at 0% FCS plus D2B+ cross-linking (d, h, n, r, v). **B.** Mean values±SD of colonies counted in three independent experiments performed in triplicate with the indicated PC3-PSMA. Statistical analysis was performed comparing results with the group control **C.** Immunoblotting of crude lysates of PC3-PSMA cells wild type (wt) or siRNA silenced for beta1, FLNa, or p130CAS. Activity of relative negative or positive probes is indicated.

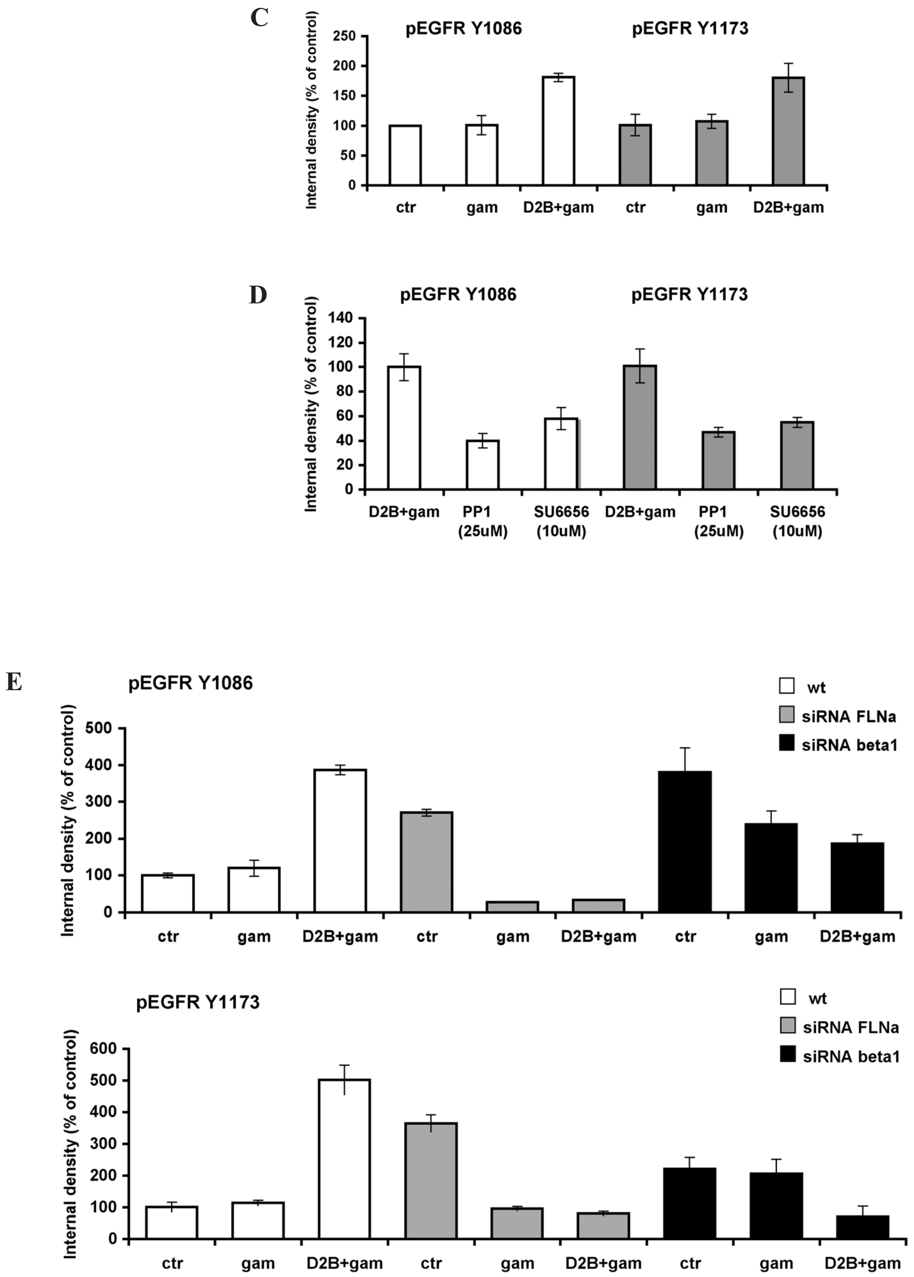

Supplementary Figure S4: Pixel densitometry of blots shown in Figure 4 (panel C, D and E). Mean values± SD of $pEGFR^{Y1086}$ or $pEGFR^{Y1173}$ observed upon PSMA cross-linking (Figure 4C) upon PSMA cross-linking performed in the presence of c-Scr inhibitor PP1 or SU6656 (Figure 4D) or in cells silenced for FLNa or beta1 (Figure 4E).

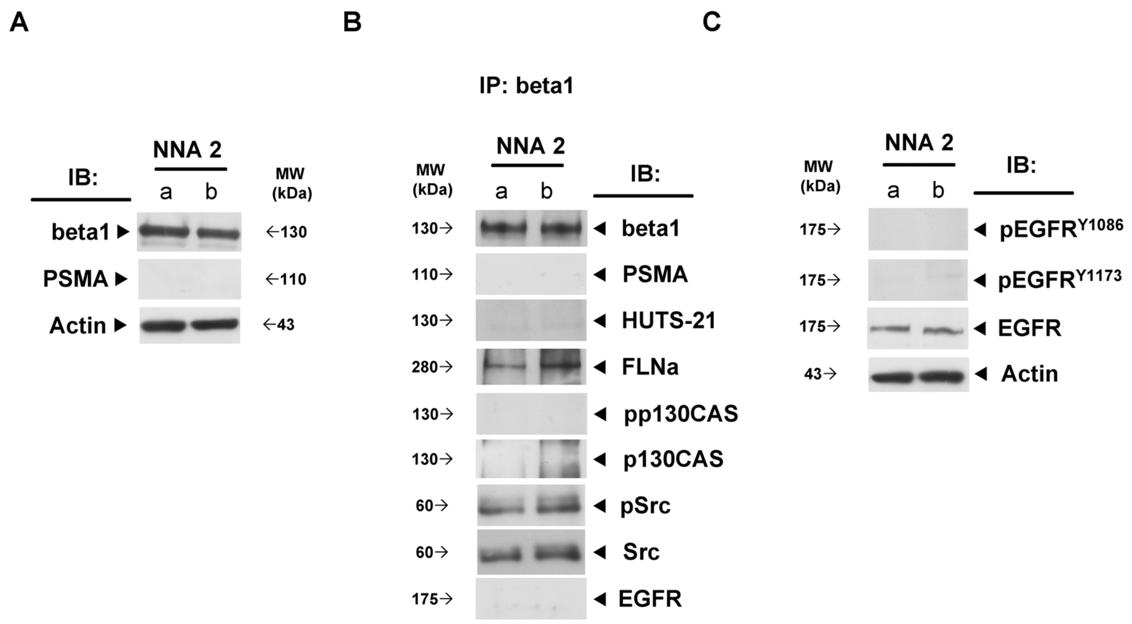

Supplementary Figure S5: NNA2 prostate specimen. A. Input of cell lysate of a and b fragments used for Immunoprecipitation. **B.** Beta 1 immunoprecipitation showing the various components of the complex. **C.** Expression of EGFR, pEGFR^{Y1086} or pEGFR^{Y1173}. Actin ensured equal loading in A and C. Proteins detected and MW are indicated.

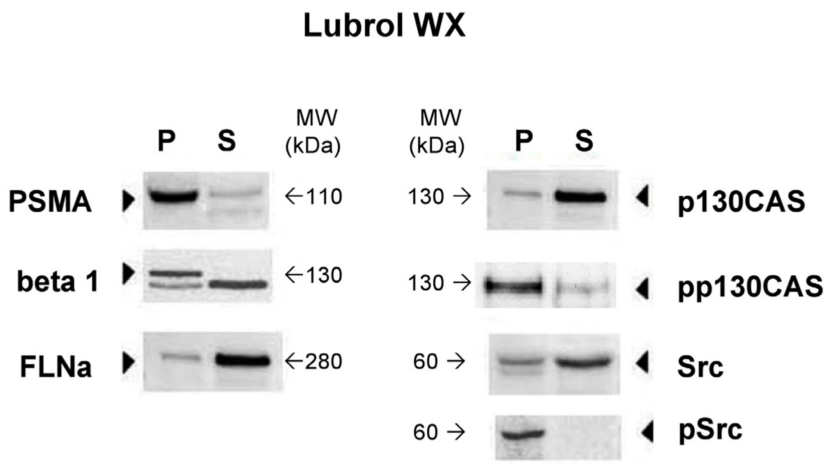

Supplementary Figure S6: Immunoblotting of soluble (S) and insoluble (P) Lubrol WX fractions of LNCaP cell lysates. Arrows indicate PSMA, beta1, FLNa, p130CAS, phospho-p130CAS, c-Src and phospho-cSrc location in the lanes. The experiment is representative of three independent assays performed with independent cell lysates.
